# Radioiodine Accumulation in a Giant Ovarian Cystadenofibroma Detected Incidentally by 131-I Whole Body Scans

**DOI:** 10.1155/2012/295617

**Published:** 2012-10-18

**Authors:** Mohammed Mebarki, Abdelghani Menemani, Abdelkader Medjahedi, Fouad Boualou, Abdelhak Slama, Sarah Ouguirti, Fatima Zahra Kherbouche, Nécib Berber

**Affiliations:** ^1^Faculty of Medicine, University of Tlemcen, Tlemcen 13000, Algeria; ^2^Department of Nuclear Medicine, University Hospital of Tlemcen, Tlemcen 13000, Algeria; ^3^Department of Surgery “A”, University Hospital of Tlemcen, Tlemcen 13000, Algeria

## Abstract

Ovarian cystadenofibroma is a relatively rare tumor; it is usually asymptomatic and is found incidentally. We present the case of a 24-year-old female patient, who had undergone total thyroidectomy for thyroid papillary carcinoma, with an asymptomatic giant cystadenofibroma, incidentally discovered by diagnostic ^131^I-SPECT/CT WBSs. We summarize the clinical history, imaging data, and histopathological study on a rare case of radioiodine accumulation in cystadenofibroma, and we discuss the mechanism of uptake of radioiodine in this case.

## 1. Introduction

Whole-body scans (WBSs) after administration of diagnostic or therapeutic doses of ^131^I have a high sensitivity in detecting physiological and/or pathological uptake of radioiodine [1]. However, several unusual lesions can cause false-positive results [2, 3]. One of the main limitations of two dimensional planar ^131^I-WBSs imaging is its lack of anatomical details. Single photon emission computed tomography/computed tomography (SPECT/CT) can correctly localize and characterize lesions presenting radioiodine uptake [4]. 

We present a rare case, an incidental finding of cystadenofibroma with radioiodine accumulation on follow-up diagnostic ^131^I-WBSs with SPECT/CT.

## 2. Case Presentation

A 24-year-old female patient underwent total thyroidectomy with central and bilateral lymphadenectomy at the age of 21 years, histopathology revealed papillary thyroid carcinoma and positive lymph nodes.

She was treated with 5.55 GBq radioiodine (^131^I) when the serum thyroid-stimulating hormone level was 100 *μ*IU/mL, and the serum thyroglobulin concentration was 68.7 ng/mL.

**Figure 1:**
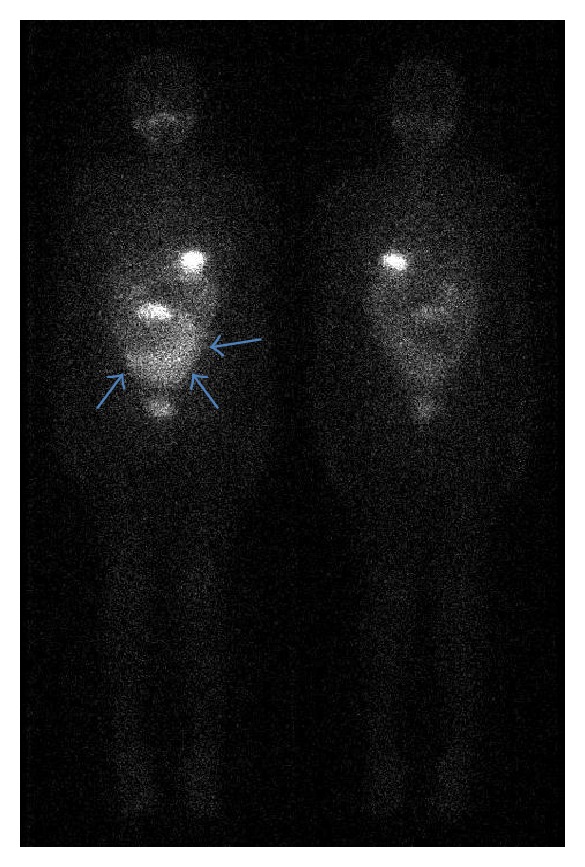
A 24-year-old female patient who had undergone total thyroidectomy with central and bilateral neck dissection for papillary thyroid cancer treated with radioiodine after. Anterior and posterior whole-body planar imaging (left and right) obtained 72 hours after oral administration of a diagnostic dose of 740 MBq of ^131^I. They show a large rounded focus of activity in the lower abdomen and pelvis. There is no abnormal radioiodine uptake in the neck areas.

Follow-up diagnostic ^131^I-WBS, 72 hours after oral administration of 370 MBq of ^131^I, performed on a gamma camera equipped with a high-energy all-purpose parallel-hole collimator (GE Infinia Hawkeye 4 slices), revealed a large rounded focus of radioiodine accumulation in the lower abdomen and pelvis. No abnormal radioiodine uptake was seen in the neck region (Figure 1).

**Figure 2:**
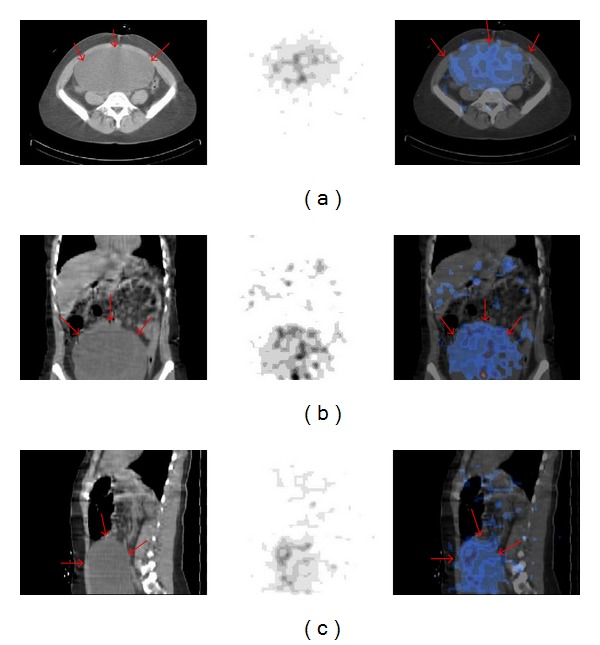
Abdominal SPECT-CT image (left, CT; middle, SPECT; right, ^131^I-SPECT/CT) revealed a very large abdominal mass with radioiodine accumulation in the transaxial (a) coronal (b), and sagittal (c) images. Note the heterogeneous distribution of radioiodine throughout the tumour mass.

Low-dose-integrated SPECT-CT of the abdomen and pelvis was then performed on the same gamma camera demonstrating a very large abdominal mass in the transverse coronal and sagittal images with heterogeneous distribution of ^131^I throughout the tumour mass (Figure 2). 

At the time of diagnostic ^131^I-WBS the serum TSH level was 100 *μ*IU/mL and the serum thyroglobulin concentration was 20 ng/mL.

Gynaecological anamnesis reported menarche appeared at the age of 14, regular menstrual cycles, a two-month history of swelling in the lower abdomen, gradual abdominal enlargement, discomfort, and urinary compressive symptoms; these clinical manifestations were neglected by the patient.

**Figure 3:**
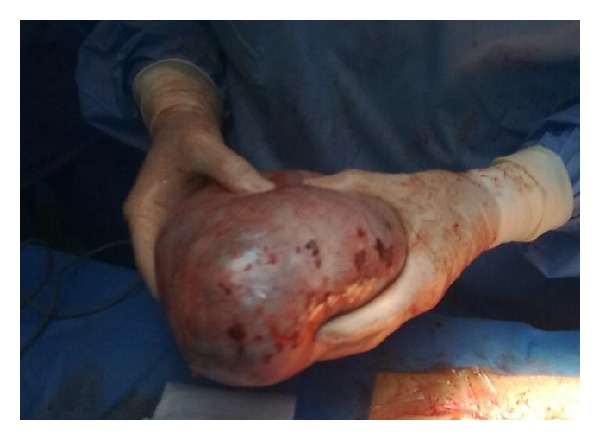
Gross photograph surgical specimen shows tumor, measuring 19 × 17 × 16 cm in the left ovary.

One month later, laparotomy was performed and the ovarian mass, measuring approximately 19 × 17 × 16 cm, was completely resected (Figure 3). Histopathological analysis revealed a borderline serous ovarian cystadenofibroma, the existence of thyroid tissue was excluded. The patient is well on followup.

## 3. Discussion

This common benign ovarian tumor is frequently incidentally discovered in patients without clinical evidence. We describe an unusual case of incidental detection of such tumors in a ^131^I-WBS with SPECT-CT.

Previous incidental findings of benign and malignant ovarian tumors on planar whole body scan have been described in the literature [5–9]. To our knowledge, only two previous cases of radioiodine uptake in a cystadenofibroma have been reported until March 2012. Flug et al. have reported a case of radioiodine accumulation in a large adnexal cystadenofibroma [10]. Song et al. have reported a case of false-positive ^131^I uptake by an ovarian serous cystadenofibroma [11].

The mechanism of radioiodine accumulation in this ovarian tumor is still poorly understood. We propose two possible theories, one based on the nonspecific radioiodine accumulation in inflamed tissues, the other based on the active transport of radioiodine due to the presence of the sodium iodide symporter (NIS) in the tumor cells [10, 11].

In conclusion, a wider use of SPECT/CT in the followup of patients with differentiated thyroid carcinoma is suggested, particularly when planar ^131^I-WBSs is not conclusive.

The presence of abnormal pelvic uptake of radioiodine in WBSs needs a gynecological examination and radiological exploration [12, 13].

Patient clinical information and prior radiological investigations are crucial elements for WBSs interpretation.
